# Supplementary material for: The state of artificial intelligence in medical research: A survey of corresponding authors from top medical journals
Source: PLoS One. 2024 Aug 23;19(8):e0309208. doi: 10.1371/journal.pone.0309208 (PMC11343420; doi:10.1371/journal.pone.0309208)
Supplement: S1 Appendix — (DOCX) [file pone.0309208.s001.docx]

**S1 File**

**Survey**

Survey Questionnaire

| **Q1. I confirm that I was a corresponding author in 2022 for a medical article in one of the following journals: (Journals list)** |
| --- |
|  |
| **Q2. What is your age?** |
| < 35 |
| 35 to 44 |
| 45 to 54 |
| 55 to 64 |
| 65 or older |
|  |
| **Q3. What is your gender?** |
| Female |
| Male |
| None of the above |
| I prefer not to answer |
|  |
| **Q4. Which is your current role?** |
| Physician |
| Researcher - Academic |
| Researcher - Clinician |
| Researcher - Other |
| Journal Editor |
| Student |
| Other (specify) |
|  |
| **Q5. In which country do you primarily work?** |
| (countries list) |
|  |
| **Q6. When was your first medical article published?** |
| < 5 years |
| 5 - 15 years |
| > 15 years |
|  |
| **Q7. Which is your scopus H-index?** |
| (open answer) |
|  |
| **Q8. How familiar are you with the concept and tools of AI?** |
| Not at all |
| Little |
| Moderate |
| Quite |
| Extensively |
|  |
| **Q9. Have you received any training specifically for using AI in Scientific Production?** |
| Yes |
| No |
| I don't know |
|  |
| **Q10. Have you ever used any kind of Artificial Intelligence in Scientific Production?** |
| Yes |
| No |
|  |
| **Q11. What AI have you used in Scientific Production?** |
| GPT |
| Google Bard |
| Microsoft Bing |
| Others LLM |
| Image Generators (such as DALL-E) |
| Database Research Assistant (such as Elicit) |
| Meta-Analysis Assistant |
| Other (please specify) |
|  |
| **Q12. If you have used any ChatBot (such as GPT), how did you employ it?** |
| Proofreading |
| Selecting related articles |
| Writing draft |
| Generating new ideas |
| Synthesizing information |
| Data managing |
| Translation into another language |
| Rephrasing |
| Other (please specify) |
|  |
| **Q13. Have you ever used a ChatBot for scientific production without citing it in the acknowledgments?** |
| Yes |
| No |
| Not Applicable |
|  |
| **Q14. Will you use any kind of Artificial Intelligence in Scientific Production in the future?** |
| Higly improbable |
| Improbable |
| I don't know |
| Probable |
| Surely |
|  |
| **Q15. Do you think it is possible to recognize a text written by a human from one entirely written by an AI?** |
| Always |
| Sometimes |
| I don't know |
| Rarely |
| Never |
|  |
| **Q16. Do you think that Artificial Intelligence, in general, can improve the quality of scientific articles?** |
| Completely agree |
| Agree |
| I don't know |
| I disagree |
| I completely disagree |
|  |
| **Q17. Will AI replace the role of medical researchers in the future?** |
| Completely agree |
| Agree |
| I don't know |
| Disagree |
| Completely disagree |
|  |
| **Q18. What are the primary critical factors to consider when utilizing AI in scientific production?** |
| Technical Problems (slow performance, bugs..) |
| Costs |
| Content Errors (Artificial Intelligence Hallucinations) |
| Ethical Aspects |
| Privacy Aspects |
| Other (please specify) |
|  |
| **Q19. What area of medicine do you believe will benefit the most from the application of AI?** |
| Drug Development |
| Diagnosis |
| Treatment |
| Telemedicine |
| Automated Radiographic Report Generation |
| Big Data Management |
| Other (please specify) |
|  |
| **Q20. To what extent will the work of a clinical physician be replaced by the advent of AI in the next 20 years?** |
| Completely |
| Mostly |
| Partially |
| Nothing will change |
| I don't know |
