# Supplementary material for: The state of artificial intelligence in medical research: A survey of corresponding authors from top medical journals
Source: PLoS One. 2024 Aug 23;19(8):e0309208. doi: 10.1371/journal.pone.0309208 (PMC11343420; doi:10.1371/journal.pone.0309208)
Supplement: S2 Appendix — (DOCX) [file pone.0309208.s002.docx]

**S2 File: List of the Leading 15 Impact Factor Medical Journals**

1. Nejm (The New England Journal of Medicine)
2. The Lancet
3. Jama (Journal of the American Medical Association)
4. Nature Medicine
5. Bmj (British Medical Journal)
6. CA-A Cancer J for Clinicians
7. Nature Reviews Drug Discovery
8. Nature Reviews Immunology
9. Lancet Microbe
10. Nature Reviews Disease Primers
11. Nature Reviews Cancer
12. Nature Reviews Clinical Oncology
13. World Psychiatry
14. Lancet Respiratory Medicine
15. Nature Reviews Genetics
