# Supplementary material for: The state of artificial intelligence in medical research: A survey of corresponding authors from top medical journals
Source: PLoS One. 2024 Aug 23;19(8):e0309208. doi: 10.1371/journal.pone.0309208 (PMC11343420; doi:10.1371/journal.pone.0309208)
Supplement: Attachment — Submitted filename: review.docx [file pone.0309208.s003.docx]

This research entitled as “The state of AI in medical research: a survey of corresponding authors from top medical journals” has the following points to be addressed:

1. Abstract need to be concise and elaborate it clearly.
2. Title is not fully defended in the Abstract. Either change the title of the manuscript of explain it in the abstract.
3. Introduction part is also missing the context of the title i.e. AI in medical field is not fully defined.
4. The result phase showing the survey is related to scientific production and language translation etc. whether title showing the medical field.
5. Literature related to the field is not well organized.
6. Environmental concerns, problem statements and solution, future directions in the conclusion is missing.
