# Supplementary material for: The state of artificial intelligence in medical research: A survey of corresponding authors from top medical journals
Source: PLoS One. 2024 Aug 23;19(8):e0309208. doi: 10.1371/journal.pone.0309208 (PMC11343420; doi:10.1371/journal.pone.0309208)
Supplement: Attachment — Submitted filename: response to reviewers-2.docx [file pone.0309208.s004.docx]

**Response to Reviewer #1**

**Q1. Abstract need to be concise and elaborate it clearly.**

We have revised the abstract to be both concise and more explicit in its presentation.

**Q2. Title is not fully defended in the Abstract. Either change the title of the manuscript or explain it in the abstract.**

The Title now states: “*The state of Artificial Intelligence in medical research: a survey of corresponding authors from top medical journals*”.

We also change the abstract.

**Q3. Introduction part is also missing the context of the title i.e., AI in the medical field is not fully defined.**

“AI in medical research” has been explained in the introduction.

**Q4. The result phase showing the survey is related to scientific production and language translation etc. whether title showing the medical field.**

The title has been adapted with “medical research”.

**Q5. Literature related to the field is not well organized.**

We have updated the literature review.

**Q6. Environmental concerns, problem statements, and solution, future directions in the conclusion is missing.**

Environmental concerns are a significant issue, and we have addressed this by adding a section on the topic. We have also incorporated the requested information as needed.

**Response to Reviewer #2**

**1. Suggested Modification to the Title of the Manuscript and avoid short form in abstract e.g. AI.**
We have revised the title to better reflect the focus of our study and expanded the term "AI" to "Artificial Intelligence" in the abstract to improve clarity.

**2. It is recommended to avoid the use of first-person pronouns (such as "I," "we," and "our") throughout the manuscript.**

That’s a fair point. We have edited the manuscript to eliminate first-person pronouns, adopting a more objective and impersonal tone throughout.

**3. The abstract provides a concise summary of the manuscript, mentioning the purpose, methodology, and key findings of the survey. However, it could be enhanced by including more specific details about the results, such as the percentage of respondents familiar with AI and the specific tasks they integrated AI technologies into. Additionally, consider including a sentence highlighting the main implications or recommendations arising from the findings.**

The abstract has been enhanced with specific details about the results, including the percentage of respondents familiar with AI. We have also added a sentence to summarize the main implications and recommendations of our findings.

**4. The introduction provides a clear rationale for conducting the survey and highlights the importance of understanding the role of AI in medical research. However, it could benefit from a more comprehensive review of the existing literature on AI in medical research.**

A bibliography has been updated with a more comprehensive review.

**5. Also, include key studies, advancements, or challenges in the field to provide a stronger context for the survey. Additionally, consider discussing the potential impact of AI in terms of improving patient outcomes, accelerating research processes, or addressing specific medical challenges.**
The bibliography has been updated with a more recent review.

**6. The methodology section provides a general overview of the survey design and data collection process. However, it would greatly benefit from more detailed information. Include the specific survey questions like the integration of AI technologies, the challenges faced by researchers, etc.**
The specific survey questions have been added in the appendix as a supplement.

**7. Additionally, provide information on the response rate, demographics of the respondents, and any measures taken to ensure the representativeness of the sample. Including these details will increase the transparency and rigor of the study.**

More information has been provided to increase the study's transparency.

**8. The results section presents the key findings of the survey, but it lacks in-depth analysis and interpretation. Consider providing more specific data, such as the percentage or frequency of responses for each survey question, to support the findings. Additionally, analyze the results in relation to the research questions or hypotheses posed at the beginning of the manuscript.**

More information on these issues has been provided to increase the study's transparency.

**9. Provide a more nuanced discussion of the variations in responses based on different factors, such as geographic location, years of experience, or journal impact factor. This will help readers better understand the implications and limitations of the findings.**

The discussion verts on variations in responses based on different factors.

**10. The discussion section should include the main findings and provide a comprehensive analysis. Start by reiterating the key findings and their significance. Then, delve into a more detailed discussion of the advantages, drawbacks, and challenges associated with integrating AI technologies in medical research, as mentioned in the abstract.**

A detailed discussion of the advantages, drawbacks, and challenges associated is provided.

**11. Include specific examples or case studies to illustrate the potential applications of AI and highlight the ethical considerations, bias mitigation strategies, and cyber security measures that need to be addressed.**

It is not in the scope of our paper.

**12. Discuss how this survey's findings align with or contribute to the existing literature on AI in medical research.**

A section detailing the contribution of this survey to the existing body of literature on AI in medical research is included.

**13. Finally, provide clear recommendations for future research, policy changes, or guidelines to support the effective and ethical use of AI in the field.**

The section on ethical guidelines have been revised.

**14. In conclusion, consider reiterating the key recommendations or action points mentioned in the discussion section. Emphasize the potential benefits of integrating AI technologies in medical research while acknowledging the challenges and the need for further research and development.**
The conclusions have been rewritten.

**15. Avoid introducing new information or ideas in the conclusion.**

No new information has been introduced in the conclusion.

**16. Ensure that all references cited in the manuscript are included in the reference section and formatted correctly according to the journal's guidelines. Additionally, consider expanding the list of references to include recent and relevant studies on AI in medical research.**
This has been done, as requested.

We hope these comprehensive responses address the concerns raised and significantly enhance the manuscript's clarity and impact. Thank you for your valuable feedback.
