# Supplementary material for: The state of artificial intelligence in medical research: A survey of corresponding authors from top medical journals
Source: PLoS One. 2024 Aug 23;19(8):e0309208. doi: 10.1371/journal.pone.0309208 (PMC11343420; doi:10.1371/journal.pone.0309208)
Supplement: Attachment — Submitted filename: Reviewer.docx [file pone.0309208.s005.docx]

Reviewer #3:

**Q1: Your abstract contains too much background information. A good abstract should briefly and clearly convey the impact of your research without unnecessary length. Here are some key points to improve your abstract:**

**i. Context and Specific Topic: Include a sentence that briefly describes the general topic and the specific topic of your**

**research. This helps set the stage for your study without delving into excessive background details.**

**ii. Central Question or Problem: Clearly state the central questions or problems your research addresses. Explain why**

**these questions are important. Are you examining a new topic, filling a gap in previous research, applying new**

**methods to existing data, or resolving a dispute in the literature?**

**iii. Research Methods: Summarize your research and/or analytical methods. This should be brief but informative enough to give readers an understanding of how you conducted your study.**

**iv. Main Findings: Highlight your main findings, results, or arguments. Focus on the most significant outcomes of your research.**

**v. Significance and Implications: Explain the significance or implications of your findings. Why do these results matter? How do they contribute to your field of study?**

A1: We thank the Reviewer for the time spent reviewing our article. We have attempted to shorten the introduction as per your suggestion. However, your first comment contradicts the feedback provided in the last round of revisions by Reviewer #2 (point 3). If further changes are required, we kindly request guidance from the Editor, as we are encountering conflicting comments.

**Q2:II- At the end of the introduction add a paragraph to describe the next sections. For example: The rest of the paper is organized as follows: Section 2 presents the method, Section 3 ....**

A2: We respectfully disagree with the Reviewers. It is standard practice in medical publishing to include methods, results, and discussion sections, making an additional descriptive paragraph unnecessary. Furthermore, research article introductions typically conclude with the study's main objective, as seen in our study, where it states:”To address this, the authors designed a survey to examine the level of familiarity, understanding, and perspectives among contributing authors in premier medical journals regarding the role and impact of artificial intelligence in top scientific research and authorship. We hypothesize that, given the novelty of large language models (LLMs), researchers might not be familiar with their use and may not have implemented them in their daily practice.”

**Q3: Add a related work section after the introduction to show what others have done.**

A3: It is the first work on this topic, for this reason there is not other literature on the topic. We tried to discuss at our best in the discussion section. If Reviewer believe that we missed some important information we would be delighted to further improve our discussion

**Q4- What hypothesis you are trying to verify or what research questions you are answering. You have to clearly state this in the paper.**

A4: We added our hypothesis at the end of the introduction that now reads:”To address this, the authors designed a survey to examine the level of familiarity, understanding, and perspectives among contributing authors in premier medical journals regarding the role and impact of artificial intelligence in top scientific research and authorship. We hypothesize that, given the novelty of large language models (LLMs), researchers might not be familiar with their use and may not have implemented them in their daily practice.”

**Q5: The survey start collection date is July 12 or 13? In the methods you have stated 13 while in the results 12.**

A5: July 13. Thank you for pointing out this typo.

**Q6:The results of statistical analysis described in the methodology section are not discussed in the results.**

A6: You are absolutely right and we removed the statistical analysis not used in ourresearch article

**Q7: Fix paper spacings for example in sentence two in the introduction "The giant leapthese ..." must be "The giant leap these...". This issue happens through out all the paper.**

A7: We would like to apologize with the Reviewer and we believe it could have been related to a compatibility problem among different software. We hope that now it is resolved

**Q8: The conclusion is too short, you have to briefly discuss numbers from your findings. The conclusion of a research paper restates the research problem and summarizes your arguments or findings.**

A8: We reformulated the conclusion as follows:

“**CONCLUSIONS**

This survey revealed varying degrees of familiarity with AI tools among researchers, with many in high-impact journals beginning to integrate AI into their work. The majority of respondents were from the USA and UK, with 54.1% from English-speaking countries. Only 5.7% indicated extensive familiarity with AI, and 24% used AI tools in scientific content creation, predominantly ChatGPT. Despite low training rates in AI (less than 1%), its use is gradually becoming more prevalent in scientific research and authorship.

“

**Q9: The appendix you referred to in the paper was not attached with the submission.**

A9:Provided
